# 2-Ferrocenyl-6-methyl­pyridin-3-ol

**DOI:** 10.1107/S1600536808039597

**Published:** 2008-11-29

**Authors:** Zhi-Qiang Wang, Chen Xu, Fei-Fei Cen, Ying-Fei Li, Bao-Ming Ji

**Affiliations:** a College of Chemistry and Chemical Engineering, Luoyang Normal University, Luoyang 471022, People’s Republic of China; b Chemical Engineering and Pharmaceutics School, Henan University of Science and Technology, Luoyang 471003, People’s Republic of China

## Abstract

In the title compound, [Fe(C_5_H_5_)(C_11_H_10_NO)], the dihedral angle between the pyridyl and substituted cyclo­penta­dienyl rings is 20.4 (3)°. The H atoms of the methyl group are disordered over two positions; their site-occupation factors were fixed at 0.5. The crystal structure is stabilized by well defined inter­molecular O—H⋯N and C—H⋯O hydrogen bonds, leading to the formation of a two-dimensional network parallel to (101).

## Related literature

For ferrocene and its derivatives, see: Beletskaya *et al.* (2001 ▶); Hayashi & Togni (1995 ▶); Kealy & Pauson (1951 ▶); Sarhan & Izumi (2003 ▶); Staveren & Metzler-Nolte (2004 ▶); Xu *et al.* (2007 ▶).
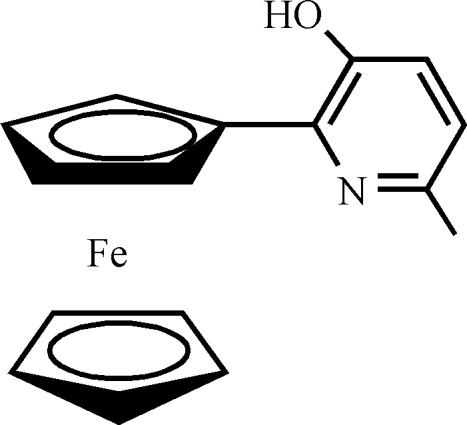


## Experimental

### 

#### Crystal data

[Fe(C_5_H_5_)(C_11_H_10_NO)]
*M* *_r_* = 293.14
Monoclinic, 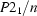

*a* = 10.4370 (13) Å
*b* = 12.7196 (15) Å
*c* = 10.5424 (13) Å
β = 111.0330 (10)°
*V* = 1306.3 (3) Å^3^
*Z* = 4
Mo *K*α radiation
μ = 1.14 mm^−1^
*T* = 291 (2) K
0.37 × 0.23 × 0.21 mm

#### Data collection

Bruker SMART APEX CCD diffractometer
Absorption correction: multi-scan (*SADABS*; Sheldrick, 1996 ▶) *T*_min_ = 0.675, *T*_max_ = 0.793
7569 measured reflections
2422 independent reflections
1970 reflections with *I* > 2σ(*I*)
*R*_int_ = 0.021

#### Refinement

*R*[*F* ^2^ > 2σ(*F* ^2^)] = 0.037
*wR*(*F* ^2^) = 0.099
*S* = 1.08
2422 reflections
173 parameters
H-atom parameters constrained
Δρ_max_ = 0.63 e Å^−3^
Δρ_min_ = −0.54 e Å^−3^

### 

Data collection: *SMART* (Bruker, 2004 ▶); cell refinement: *SAINT* (Bruker, 2004 ▶); data reduction: *SAINT*; program(s) used to solve structure: *SHELXS97* (Sheldrick, 2008 ▶); program(s) used to refine structure: *SHELXL97* (Sheldrick, 2008 ▶); molecular graphics: *SHELXTL* (Sheldrick, 2008 ▶); software used to prepare material for publication: *SHELXTL*.

## Supplementary Material

Crystal structure: contains datablocks global, I. DOI: 10.1107/S1600536808039597/fj2173sup1.cif

Structure factors: contains datablocks I. DOI: 10.1107/S1600536808039597/fj2173Isup2.hkl

Additional supplementary materials:  crystallographic information; 3D view; checkCIF report

## Figures and Tables

**Table 1:** Hydrogen-bond geometry (Å, °)

*D*—H⋯*A*	*D*—H	H⋯*A*	*D*⋯*A*	*D*—H⋯*A*
O1—H1⋯N1^i^	0.82	1.96	2.774 (3)	169
C7—H7⋯O1	0.98	2.39	2.866 (4)	109

Symmetry code: (i) 
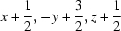
.

**Fig. 1.**
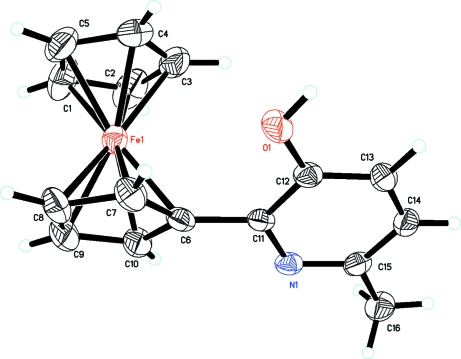
The molecular structure of the title compound with displacement ellipsoids at the 30% probability level. Only one disordered component of the methyl group is shown.

**Fig. 2.**
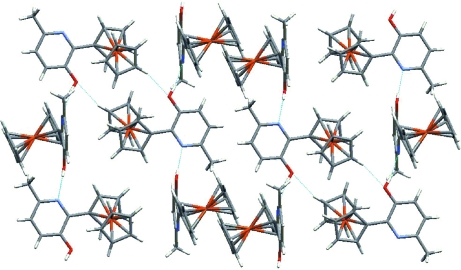
Partial view of the crystal packing showing the intermolecular O—H···N and C—H···O hydrogen bonds. One disordered component of the methyl group has been omitted for clarity.

## supplementary crystallographic information

### Comment

Since the discovery of ferrocene in the 1950's (Kealy & Pauson, 1951), the fascinating structural properties of ferrocene and its derivatives have been the subject of increasing interest in all fields of organometallic chemistry (Hayashi *et al.*, 1995; Staveren *et al.*, 2004; Xu *et al.*, 2007). Among them, ferrocene-heterocycles are one of the most important ones (Sarhan & Izumi, 2003). Herein we report the crystal structure of the title compound.

A view of the molecular structure of the title compound is given in Fig.1. The hydrogen atoms of methyl groups are disordered; site-occupation factors were fixed at 0.5. The pyridyl and Cp ring form a dihedral angle of 20.4 (3)°. In the crystal of the title compound, intermolecular O—H···N and C—H···O hydrogen bonds are present(Table 1), resulting in a two-dimensional supramolecular architecture(Fig.2).

### Experimental

The title compound was prepared as described in literature (Beletskaya *et al.*, 2001) and recrystallized from dichloromethane-petroleum ether solution at room temperature to give the desired product as red crystals suitable for single-crystal X-ray diffraction.

### Refinement

H atoms attached to C atoms of the title compound were placed in geometrically idealized positions and treated as riding with C—H distances constrained to 0.93–0.96 Å, and with *U*ĩso~(H)=1.2Ueq(C)(1.5Ueq for methyl H).

### Crystal data

**Table:**

[Fe(C_5_H_5_)(C_11_H_10_NO)]	*F*_000_ = 608
*M**_r_* = 293.14	*D*_x_ = 1.491 Mg m^−^^3^
Monoclinic, *P*2_1_/*n*	Mo *K*α radiation λ = 0.71073 Å
Hall symbol: -P 2yn	Cell parameters from 2766 reflections
*a* = 10.4370 (13) Å	θ = 2.4–25.0º
*b* = 12.7196 (15) Å	µ = 1.14 mm^−^^1^
*c* = 10.5424 (13) Å	*T* = 291 (2) K
β = 111.0330 (10)º	Block, red
*V* = 1306.3 (3) Å^3^	0.37 × 0.23 × 0.21 mm
*Z* = 4	

### Data collection

**Table:**

Bruker SMART APEX CCD diffractometer	2422 independent reflections
Radiation source: fine-focus sealed tube	1970 reflections with *I* > 2σ(*I*)
Monochromator: graphite	*R*_int_ = 0.021
*T* = 291(2) K	θ_max_ = 25.5º
φ and ω scans	θ_min_ = 2.4º
Absorption correction: multi-scan(SADABS; Sheldrick, 1996)	*h* = −10→12
*T*_min_ = 0.675, *T*_max_ = 0.794	*k* = −15→15
7569 measured reflections	*l* = −12→12

### Refinement

**Table:**

Refinement on *F*^2^	Secondary atom site location: difference Fourier map
Least-squares matrix: full	Hydrogen site location: inferred from neighbouring sites
*R*[*F*^2^ > 2σ(*F*^2^)] = 0.037	H-atom parameters constrained
*wR*(*F*^2^) = 0.099	*w* = 1/[σ^2^(*F*_o_^2^) + (0.046*P*)^2^ + 0.7167*P*] where *P* = (*F*_o_^2^ + 2*F*_c_^2^)/3
*S* = 1.09	(Δ/σ)_max_ = 0.001
2422 reflections	Δρ_max_ = 0.63 e Å^−^^3^
173 parameters	Δρ_min_ = −0.54 e Å^−^^3^
Primary atom site location: structure-invariant direct methods	Extinction correction: none

### Special details

**Table:**

Geometry. All e.s.d.'s (except the e.s.d. in the dihedral angle between two l.s. planes)are estimated using the full covariance matrix. The cell e.s.d.'s are takeninto account individually in the estimation of e.s.d.'s in distances, anglesand torsion angles; correlations between e.s.d.'s in cell parameters are onlyused when they are defined by crystal symmetry. An approximate (isotropic)treatment of cell e.s.d.'s is used for estimating e.s.d.'s involving l.s. planes.
Refinement. Refinement of *F*^2^ against ALL reflections. The weighted *R*-factor *wR* andgoodness of fit *S* are based on *F*^2^, conventional *R*-factors *R* are basedon *F*, with *F* set to zero for negative *F*^2^. The threshold expression of*F*^2^ > σ(*F*^2^) is used only for calculating *R*-factors(gt) *etc*. and isnot relevant to the choice of reflections for refinement. *R*-factors basedon *F*^2^ are statistically about twice as large as those based on *F*, and *R*-factors based on ALL data will be even larger.

### Fractional atomic coordinates and isotropic or equivalent isotropic displacement parameters (Å^2^)

**Table:**

	*x*	*y*	*z*	*U*_iso_*/*U*_eq_	Occ. (<1)
Fe1	0.35569 (4)	1.02041 (3)	0.20685 (4)	0.04595 (16)	
O1	0.5672 (2)	0.78281 (18)	0.36235 (18)	0.0576 (5)	
H1	0.6489	0.7688	0.3993	0.086*	
N1	0.3444 (2)	0.74252 (16)	0.0124 (2)	0.0424 (5)	
C1	0.3813 (5)	1.1764 (3)	0.1792 (5)	0.0962 (14)	
H1A	0.3075	1.2283	0.1454	0.115*	
C2	0.4350 (5)	1.1151 (3)	0.0982 (4)	0.0905 (13)	
H2	0.4057	1.1177	−0.0011	0.109*	
C3	0.5377 (4)	1.0523 (3)	0.1836 (4)	0.0788 (11)	
H3	0.5932	1.0019	0.1552	0.095*	
C4	0.5481 (4)	1.0718 (3)	0.3185 (4)	0.0827 (11)	
H4	0.6125	1.0386	0.4004	0.099*	
C5	0.4490 (4)	1.1502 (3)	0.3136 (4)	0.0856 (12)	
H5	0.4325	1.1806	0.3918	0.103*	
C6	0.3115 (3)	0.8626 (2)	0.1744 (3)	0.0452 (6)	
C7	0.3146 (3)	0.8949 (3)	0.3049 (3)	0.0573 (7)	
H7	0.3754	0.8666	0.3922	0.069*	
C8	0.2150 (3)	0.9752 (3)	0.2872 (4)	0.0693 (9)	
H8	0.1948	1.0114	0.3600	0.083*	
C9	0.1511 (3)	0.9943 (3)	0.1476 (4)	0.0778 (11)	
H9	0.0787	1.0462	0.1061	0.093*	
C10	0.2095 (3)	0.9257 (3)	0.0766 (3)	0.0638 (9)	
H10	0.1849	0.9227	−0.0222	0.077*	
C11	0.3946 (2)	0.78116 (19)	0.1409 (2)	0.0389 (6)	
C12	0.5209 (3)	0.7445 (2)	0.2335 (2)	0.0419 (6)	
C13	0.5943 (3)	0.6722 (2)	0.1894 (3)	0.0487 (7)	
H13	0.6780	0.6472	0.2490	0.058*	
C14	0.5436 (3)	0.6372 (2)	0.0573 (3)	0.0489 (6)	
H14	0.5936	0.5896	0.0264	0.059*	
C15	0.4175 (3)	0.6734 (2)	−0.0295 (3)	0.0455 (6)	
C16	0.3599 (3)	0.6378 (3)	−0.1758 (3)	0.0637 (8)	
H16A	0.2724	0.6705	−0.2207	0.096*	0.50
H16B	0.3490	0.5628	−0.1790	0.096*	0.50
H16C	0.4217	0.6574	−0.2206	0.096*	0.50
H16D	0.4230	0.5900	−0.1929	0.096*	0.50
H16E	0.3464	0.6977	−0.2345	0.096*	0.50
H16F	0.2737	0.6030	−0.1929	0.096*	0.50

### Atomic displacement parameters (Å^2^)

**Table:**

	*U*^11^	*U*^22^	*U*^33^	*U*^12^	*U*^13^	*U*^23^
Fe1	0.0494 (3)	0.0480 (3)	0.0396 (2)	0.00711 (17)	0.01483 (18)	−0.00218 (16)
O1	0.0420 (11)	0.0758 (14)	0.0404 (10)	0.0147 (10)	−0.0031 (8)	−0.0069 (9)
N1	0.0351 (11)	0.0414 (12)	0.0407 (11)	−0.0034 (9)	0.0014 (9)	0.0023 (9)
C1	0.143 (4)	0.044 (2)	0.120 (4)	0.011 (2)	0.070 (3)	−0.003 (2)
C2	0.150 (4)	0.059 (2)	0.088 (3)	−0.012 (2)	0.074 (3)	−0.002 (2)
C3	0.080 (3)	0.067 (2)	0.110 (3)	−0.024 (2)	0.058 (2)	−0.031 (2)
C4	0.063 (2)	0.089 (3)	0.092 (3)	−0.021 (2)	0.022 (2)	−0.033 (2)
C5	0.102 (3)	0.076 (2)	0.091 (3)	−0.020 (2)	0.050 (2)	−0.039 (2)
C6	0.0298 (13)	0.0498 (16)	0.0499 (15)	−0.0026 (11)	0.0069 (11)	0.0015 (12)
C7	0.0532 (18)	0.068 (2)	0.0575 (17)	0.0024 (15)	0.0282 (14)	0.0088 (14)
C8	0.0517 (19)	0.085 (2)	0.084 (2)	0.0068 (17)	0.0393 (19)	−0.0064 (18)
C9	0.0382 (17)	0.090 (3)	0.089 (3)	0.0194 (17)	0.0033 (18)	−0.013 (2)
C10	0.0414 (16)	0.071 (2)	0.0606 (18)	0.0121 (15)	−0.0037 (14)	−0.0132 (16)
C11	0.0315 (13)	0.0380 (13)	0.0413 (13)	−0.0045 (10)	0.0060 (11)	0.0036 (10)
C12	0.0370 (14)	0.0419 (14)	0.0375 (13)	−0.0012 (11)	0.0019 (11)	0.0020 (10)
C13	0.0405 (15)	0.0439 (15)	0.0489 (15)	0.0084 (12)	0.0003 (12)	0.0034 (12)
C14	0.0488 (16)	0.0385 (14)	0.0525 (15)	0.0045 (12)	0.0097 (13)	−0.0019 (11)
C15	0.0464 (16)	0.0372 (14)	0.0450 (14)	−0.0056 (12)	0.0067 (12)	−0.0005 (11)
C16	0.063 (2)	0.0625 (19)	0.0502 (17)	0.0023 (16)	0.0019 (15)	−0.0102 (14)

### Geometric parameters (Å, °)

**Table:**

Fe1—C8	2.024 (3)	C6—C7	1.426 (4)
Fe1—C9	2.025 (3)	C6—C10	1.433 (4)
Fe1—C7	2.029 (3)	C6—C11	1.473 (4)
Fe1—C2	2.032 (4)	C7—C8	1.421 (4)
Fe1—C4	2.036 (3)	C7—H7	0.9800
Fe1—C1	2.036 (4)	C8—C9	1.401 (5)
Fe1—C5	2.038 (4)	C8—H8	0.9800
Fe1—C10	2.040 (3)	C9—C10	1.421 (5)
Fe1—C3	2.041 (4)	C9—H9	0.9800
Fe1—C6	2.061 (3)	C10—H10	0.9800
O1—C12	1.358 (3)	C11—C12	1.408 (3)
O1—H1	0.8200	C12—C13	1.381 (4)
N1—C15	1.339 (3)	C13—C14	1.374 (4)
N1—C11	1.357 (3)	C13—H13	0.9300
C1—C5	1.377 (6)	C14—C15	1.385 (4)
C1—C2	1.412 (5)	C14—H14	0.9300
C1—H1A	0.9800	C15—C16	1.509 (4)
C2—C3	1.381 (6)	C16—H16A	0.9600
C2—H2	0.9800	C16—H16B	0.9600
C3—C4	1.408 (5)	C16—H16C	0.9600
C3—H3	0.9800	C16—H16D	0.9600
C4—C5	1.425 (6)	C16—H16E	0.9600
C4—H4	0.9800	C16—H16F	0.9600
C5—H5	0.9800		
			
C8—Fe1—C9	40.49 (16)	C1—C5—Fe1	70.2 (2)
C8—Fe1—C7	41.06 (13)	C4—C5—Fe1	69.5 (2)
C9—Fe1—C7	68.66 (15)	C1—C5—H5	126.2
C8—Fe1—C2	155.18 (17)	C4—C5—H5	126.2
C9—Fe1—C2	121.4 (2)	Fe1—C5—H5	126.2
C7—Fe1—C2	163.08 (15)	C7—C6—C10	106.6 (3)
C8—Fe1—C4	124.23 (16)	C7—C6—C11	128.5 (2)
C9—Fe1—C4	160.47 (16)	C10—C6—C11	124.9 (3)
C7—Fe1—C4	107.62 (16)	C7—C6—Fe1	68.41 (17)
C2—Fe1—C4	67.60 (19)	C10—C6—Fe1	68.77 (17)
C8—Fe1—C1	119.26 (17)	C11—C6—Fe1	127.50 (18)
C9—Fe1—C1	107.08 (18)	C8—C7—C6	108.5 (3)
C7—Fe1—C1	154.37 (15)	C8—C7—Fe1	69.29 (19)
C2—Fe1—C1	40.61 (16)	C6—C7—Fe1	70.80 (16)
C4—Fe1—C1	67.5 (2)	C8—C7—H7	125.7
C8—Fe1—C5	105.90 (16)	C6—C7—H7	125.7
C9—Fe1—C5	123.03 (16)	Fe1—C7—H7	125.7
C7—Fe1—C5	120.16 (16)	C9—C8—C7	108.2 (3)
C2—Fe1—C5	67.68 (17)	C9—C8—Fe1	69.8 (2)
C4—Fe1—C5	40.93 (16)	C7—C8—Fe1	69.66 (17)
C1—Fe1—C5	39.52 (16)	C9—C8—H8	125.9
C8—Fe1—C10	68.53 (15)	C7—C8—H8	125.9
C9—Fe1—C10	40.92 (14)	Fe1—C8—H8	125.9
C7—Fe1—C10	68.58 (14)	C8—C9—C10	108.3 (3)
C2—Fe1—C10	109.16 (17)	C8—C9—Fe1	69.71 (19)
C4—Fe1—C10	157.32 (14)	C10—C9—Fe1	70.09 (18)
C1—Fe1—C10	125.72 (18)	C8—C9—H9	125.8
C5—Fe1—C10	160.69 (16)	C10—C9—H9	125.8
C8—Fe1—C3	162.32 (18)	Fe1—C9—H9	125.8
C9—Fe1—C3	156.78 (18)	C9—C10—C6	108.3 (3)
C7—Fe1—C3	126.37 (16)	C9—C10—Fe1	68.99 (19)
C2—Fe1—C3	39.63 (17)	C6—C10—Fe1	70.33 (16)
C4—Fe1—C3	40.41 (15)	C9—C10—H10	125.9
C1—Fe1—C3	67.29 (18)	C6—C10—H10	125.9
C5—Fe1—C3	67.95 (15)	Fe1—C10—H10	125.9
C10—Fe1—C3	122.69 (14)	N1—C11—C12	120.2 (2)
C8—Fe1—C6	68.91 (13)	N1—C11—C6	116.3 (2)
C9—Fe1—C6	68.94 (13)	C12—C11—C6	123.5 (2)
C7—Fe1—C6	40.79 (11)	O1—C12—C13	122.3 (2)
C2—Fe1—C6	126.58 (13)	O1—C12—C11	118.9 (2)
C4—Fe1—C6	121.60 (15)	C13—C12—C11	118.8 (2)
C1—Fe1—C6	163.36 (16)	C14—C13—C12	119.9 (2)
C5—Fe1—C6	156.22 (16)	C14—C13—H13	120.0
C10—Fe1—C6	40.90 (11)	C12—C13—H13	120.0
C3—Fe1—C6	109.62 (13)	C13—C14—C15	119.4 (3)
C12—O1—H1	109.5	C13—C14—H14	120.3
C15—N1—C11	120.4 (2)	C15—C14—H14	120.3
C5—C1—C2	108.7 (4)	N1—C15—C14	121.3 (2)
C5—C1—Fe1	70.3 (2)	N1—C15—C16	118.0 (2)
C2—C1—Fe1	69.5 (2)	C14—C15—C16	120.7 (3)
C5—C1—H1A	125.7	C15—C16—H16A	109.5
C2—C1—H1A	125.7	C15—C16—H16B	109.5
Fe1—C1—H1A	125.7	H16A—C16—H16B	109.5
C3—C2—C1	108.0 (4)	C15—C16—H16C	109.5
C3—C2—Fe1	70.5 (2)	H16A—C16—H16C	109.5
C1—C2—Fe1	69.9 (2)	H16B—C16—H16C	109.5
C3—C2—H2	126.0	C15—C16—H16D	109.5
C1—C2—H2	126.0	H16A—C16—H16D	141.1
Fe1—C2—H2	126.0	H16B—C16—H16D	56.3
C2—C3—C4	108.5 (4)	H16C—C16—H16D	56.3
C2—C3—Fe1	69.8 (2)	C15—C16—H16E	109.5
C4—C3—Fe1	69.6 (2)	H16A—C16—H16E	56.3
C2—C3—H3	125.8	H16B—C16—H16E	141.1
C4—C3—H3	125.8	H16C—C16—H16E	56.3
Fe1—C3—H3	125.8	H16D—C16—H16E	109.5
C3—C4—C5	107.2 (4)	C15—C16—H16F	109.5
C3—C4—Fe1	70.0 (2)	H16A—C16—H16F	56.3
C5—C4—Fe1	69.6 (2)	H16B—C16—H16F	56.3
C3—C4—H4	126.4	H16C—C16—H16F	141.1
C5—C4—H4	126.4	H16D—C16—H16F	109.5
Fe1—C4—H4	126.4	H16E—C16—H16F	109.5
C1—C5—C4	107.7 (3)		
			
C8—Fe1—C1—C5	79.3 (3)	C8—Fe1—C6—C11	−160.5 (3)
C9—Fe1—C1—C5	121.6 (3)	C9—Fe1—C6—C11	155.9 (3)
C7—Fe1—C1—C5	45.4 (5)	C7—Fe1—C6—C11	−122.7 (3)
C2—Fe1—C1—C5	−119.8 (4)	C2—Fe1—C6—C11	41.8 (3)
C4—Fe1—C1—C5	−38.5 (2)	C4—Fe1—C6—C11	−42.4 (3)
C10—Fe1—C1—C5	162.7 (2)	C1—Fe1—C6—C11	76.9 (6)
C3—Fe1—C1—C5	−82.4 (3)	C5—Fe1—C6—C11	−79.0 (4)
C6—Fe1—C1—C5	−165.0 (4)	C10—Fe1—C6—C11	118.3 (3)
C8—Fe1—C1—C2	−160.9 (3)	C3—Fe1—C6—C11	0.7 (3)
C9—Fe1—C1—C2	−118.6 (3)	C10—C6—C7—C8	0.8 (3)
C7—Fe1—C1—C2	165.3 (3)	C11—C6—C7—C8	−179.3 (3)
C4—Fe1—C1—C2	81.3 (3)	Fe1—C6—C7—C8	59.2 (2)
C5—Fe1—C1—C2	119.8 (4)	C10—C6—C7—Fe1	−58.4 (2)
C10—Fe1—C1—C2	−77.4 (3)	C11—C6—C7—Fe1	121.5 (3)
C3—Fe1—C1—C2	37.4 (3)	C9—Fe1—C7—C8	−37.4 (2)
C6—Fe1—C1—C2	−45.2 (7)	C2—Fe1—C7—C8	−167.0 (5)
C5—C1—C2—C3	−0.9 (5)	C4—Fe1—C7—C8	122.3 (2)
Fe1—C1—C2—C3	−60.5 (3)	C1—Fe1—C7—C8	47.7 (5)
C5—C1—C2—Fe1	59.6 (3)	C5—Fe1—C7—C8	79.3 (3)
C8—Fe1—C2—C3	161.4 (3)	C10—Fe1—C7—C8	−81.5 (2)
C9—Fe1—C2—C3	−162.0 (2)	C3—Fe1—C7—C8	163.0 (2)
C7—Fe1—C2—C3	−39.2 (6)	C6—Fe1—C7—C8	−119.5 (3)
C4—Fe1—C2—C3	37.5 (2)	C8—Fe1—C7—C6	119.5 (3)
C1—Fe1—C2—C3	118.6 (4)	C9—Fe1—C7—C6	82.08 (19)
C5—Fe1—C2—C3	81.9 (3)	C2—Fe1—C7—C6	−47.5 (6)
C10—Fe1—C2—C3	−118.5 (2)	C4—Fe1—C7—C6	−118.27 (18)
C6—Fe1—C2—C3	−76.1 (3)	C1—Fe1—C7—C6	167.2 (4)
C8—Fe1—C2—C1	42.8 (5)	C5—Fe1—C7—C6	−161.19 (19)
C9—Fe1—C2—C1	79.4 (3)	C10—Fe1—C7—C6	37.99 (17)
C7—Fe1—C2—C1	−157.8 (5)	C3—Fe1—C7—C6	−77.6 (2)
C4—Fe1—C2—C1	−81.1 (3)	C6—C7—C8—C9	−0.8 (4)
C5—Fe1—C2—C1	−36.6 (3)	Fe1—C7—C8—C9	59.4 (3)
C10—Fe1—C2—C1	123.0 (3)	C6—C7—C8—Fe1	−60.1 (2)
C3—Fe1—C2—C1	−118.6 (4)	C7—Fe1—C8—C9	−119.4 (3)
C6—Fe1—C2—C1	165.4 (3)	C2—Fe1—C8—C9	51.6 (5)
C1—C2—C3—C4	1.0 (5)	C4—Fe1—C8—C9	163.5 (2)
Fe1—C2—C3—C4	−59.1 (3)	C1—Fe1—C8—C9	82.1 (3)
C1—C2—C3—Fe1	60.1 (3)	C5—Fe1—C8—C9	122.6 (2)
C8—Fe1—C3—C2	−153.9 (4)	C10—Fe1—C8—C9	−37.8 (2)
C9—Fe1—C3—C2	42.0 (5)	C3—Fe1—C8—C9	−170.4 (4)
C7—Fe1—C3—C2	166.8 (2)	C6—Fe1—C8—C9	−81.9 (2)
C4—Fe1—C3—C2	−119.8 (3)	C9—Fe1—C8—C7	119.4 (3)
C1—Fe1—C3—C2	−38.3 (2)	C2—Fe1—C8—C7	171.0 (3)
C5—Fe1—C3—C2	−81.2 (3)	C4—Fe1—C8—C7	−77.1 (3)
C10—Fe1—C3—C2	80.6 (3)	C1—Fe1—C8—C7	−158.5 (2)
C6—Fe1—C3—C2	124.2 (2)	C5—Fe1—C8—C7	−117.9 (2)
C8—Fe1—C3—C4	−34.1 (6)	C10—Fe1—C8—C7	81.6 (2)
C9—Fe1—C3—C4	161.8 (4)	C3—Fe1—C8—C7	−51.0 (6)
C7—Fe1—C3—C4	−73.4 (3)	C6—Fe1—C8—C7	37.56 (19)
C2—Fe1—C3—C4	119.8 (3)	C7—C8—C9—C10	0.4 (4)
C1—Fe1—C3—C4	81.5 (3)	Fe1—C8—C9—C10	59.7 (3)
C5—Fe1—C3—C4	38.6 (3)	C7—C8—C9—Fe1	−59.3 (2)
C10—Fe1—C3—C4	−159.6 (2)	C7—Fe1—C9—C8	37.9 (2)
C6—Fe1—C3—C4	−116.1 (2)	C2—Fe1—C9—C8	−157.3 (2)
C2—C3—C4—C5	−0.7 (4)	C4—Fe1—C9—C8	−44.7 (6)
Fe1—C3—C4—C5	−59.9 (3)	C1—Fe1—C9—C8	−115.3 (2)
C2—C3—C4—Fe1	59.2 (3)	C5—Fe1—C9—C8	−75.0 (3)
C8—Fe1—C4—C3	168.1 (2)	C10—Fe1—C9—C8	119.4 (3)
C9—Fe1—C4—C3	−158.4 (5)	C3—Fe1—C9—C8	172.7 (3)
C7—Fe1—C4—C3	125.9 (3)	C6—Fe1—C9—C8	81.8 (2)
C2—Fe1—C4—C3	−36.8 (2)	C8—Fe1—C9—C10	−119.4 (3)
C1—Fe1—C4—C3	−80.9 (3)	C7—Fe1—C9—C10	−81.5 (2)
C5—Fe1—C4—C3	−118.1 (4)	C2—Fe1—C9—C10	83.3 (3)
C10—Fe1—C4—C3	49.4 (5)	C4—Fe1—C9—C10	−164.1 (5)
C6—Fe1—C4—C3	83.4 (3)	C1—Fe1—C9—C10	125.3 (2)
C8—Fe1—C4—C5	−73.8 (3)	C5—Fe1—C9—C10	165.6 (2)
C9—Fe1—C4—C5	−40.3 (6)	C3—Fe1—C9—C10	53.3 (5)
C7—Fe1—C4—C5	−116.0 (3)	C6—Fe1—C9—C10	−37.6 (2)
C2—Fe1—C4—C5	81.3 (3)	C8—C9—C10—C6	0.1 (4)
C1—Fe1—C4—C5	37.2 (2)	Fe1—C9—C10—C6	59.6 (2)
C10—Fe1—C4—C5	167.5 (4)	C8—C9—C10—Fe1	−59.4 (3)
C3—Fe1—C4—C5	118.1 (4)	C7—C6—C10—C9	−0.6 (4)
C6—Fe1—C4—C5	−158.5 (2)	C11—C6—C10—C9	179.6 (3)
C2—C1—C5—C4	0.5 (5)	Fe1—C6—C10—C9	−58.7 (2)
Fe1—C1—C5—C4	59.6 (3)	C7—C6—C10—Fe1	58.1 (2)
C2—C1—C5—Fe1	−59.1 (3)	C11—C6—C10—Fe1	−121.7 (3)
C3—C4—C5—C1	0.2 (5)	C8—Fe1—C10—C9	37.4 (2)
Fe1—C4—C5—C1	−60.0 (3)	C7—Fe1—C10—C9	81.7 (2)
C3—C4—C5—Fe1	60.2 (2)	C2—Fe1—C10—C9	−116.2 (3)
C8—Fe1—C5—C1	−117.0 (3)	C4—Fe1—C10—C9	166.2 (4)
C9—Fe1—C5—C1	−76.2 (3)	C1—Fe1—C10—C9	−73.9 (3)
C7—Fe1—C5—C1	−159.1 (2)	C5—Fe1—C10—C9	−39.1 (6)
C2—Fe1—C5—C1	37.6 (3)	C3—Fe1—C10—C9	−157.9 (3)
C4—Fe1—C5—C1	118.7 (4)	C6—Fe1—C10—C9	119.6 (3)
C10—Fe1—C5—C1	−46.7 (6)	C8—Fe1—C10—C6	−82.2 (2)
C3—Fe1—C5—C1	80.6 (3)	C9—Fe1—C10—C6	−119.6 (3)
C6—Fe1—C5—C1	169.4 (3)	C7—Fe1—C10—C6	−37.89 (17)
C8—Fe1—C5—C4	124.4 (3)	C2—Fe1—C10—C6	124.2 (2)
C9—Fe1—C5—C4	165.1 (2)	C4—Fe1—C10—C6	46.6 (5)
C7—Fe1—C5—C4	82.2 (3)	C1—Fe1—C10—C6	166.5 (2)
C2—Fe1—C5—C4	−81.1 (3)	C5—Fe1—C10—C6	−158.7 (4)
C1—Fe1—C5—C4	−118.7 (4)	C3—Fe1—C10—C6	82.4 (2)
C10—Fe1—C5—C4	−165.4 (4)	C15—N1—C11—C12	−3.4 (4)
C3—Fe1—C5—C4	−38.1 (3)	C15—N1—C11—C6	175.6 (2)
C6—Fe1—C5—C4	50.7 (4)	C7—C6—C11—N1	160.0 (3)
C8—Fe1—C6—C7	−37.80 (19)	C10—C6—C11—N1	−20.3 (4)
C9—Fe1—C6—C7	−81.3 (2)	Fe1—C6—C11—N1	−109.0 (2)
C2—Fe1—C6—C7	164.5 (2)	C7—C6—C11—C12	−21.1 (4)
C4—Fe1—C6—C7	80.3 (2)	C10—C6—C11—C12	158.7 (3)
C1—Fe1—C6—C7	−160.4 (5)	Fe1—C6—C11—C12	70.0 (3)
C5—Fe1—C6—C7	43.7 (4)	N1—C11—C12—O1	−178.5 (2)
C10—Fe1—C6—C7	−118.9 (3)	C6—C11—C12—O1	2.5 (4)
C3—Fe1—C6—C7	123.4 (2)	N1—C11—C12—C13	2.5 (4)
C8—Fe1—C6—C10	81.1 (2)	C6—C11—C12—C13	−176.4 (2)
C9—Fe1—C6—C10	37.6 (2)	O1—C12—C13—C14	−179.0 (3)
C7—Fe1—C6—C10	118.9 (3)	C11—C12—C13—C14	−0.1 (4)
C2—Fe1—C6—C10	−76.6 (3)	C12—C13—C14—C15	−1.4 (4)
C4—Fe1—C6—C10	−160.8 (2)	C11—N1—C15—C14	1.9 (4)
C1—Fe1—C6—C10	−41.5 (6)	C11—N1—C15—C16	−176.7 (2)
C5—Fe1—C6—C10	162.7 (3)	C13—C14—C15—N1	0.5 (4)
C3—Fe1—C6—C10	−117.7 (2)	C13—C14—C15—C16	179.1 (3)

### Hydrogen-bond geometry (Å, °)

**Table:**

*D*—H···*A*	*D*—H	H···*A*	*D*···*A*	*D*—H···*A*
O1—H1···N1^i^	0.82	1.96	2.774 (3)	169
C7—H7···O1	0.98	2.39	2.866 (4)	109

Symmetry codes: (i) *x*+1/2, −*y*+3/2, *z*+1/2.
